# Betulin Sulfonamides as Carbonic Anhydrase Inhibitors and Anticancer Agents in Breast Cancer Cells

**DOI:** 10.3390/ijms22168808

**Published:** 2021-08-16

**Authors:** Antje Güttler, Yvonne Eiselt, Anne Funtan, Andreas Thiel, Marina Petrenko, Jacqueline Keßler, Iris Thondorf, Reinhard Paschke, Dirk Vordermark, Matthias Bache

**Affiliations:** 1 Department of Radiotherapy, Martin-Luther-University of Halle-Wittenberg, Ernst-Grube-Str. 40, D-06120 Halle/Saale, Germany; yvonne.eiselt@student.uni-halle.de (Y.E.); marina.petrenko@uk-halle.de (M.P.); jacqueline.kessler@uk-halle.de (J.K.); dirk.vordermark@uk-halle.de (D.V.); matthias.bache@uk-halle.de (M.B.); 2 Biozentrum, Martin-Luther-University of Halle-Wittenberg, Weinbergweg 22, D-06120 Halle/Saale, Germany; anne.funtan@biozentrum.uni-halle.de (A.F.); reinhard.paschke@biozentrum.uni-halle.de (R.P.); 3 Institute of Biochemistry and Biotechnology, Martin-Luther University Halle-Wittenberg, Kurt-Mothes-Str. 3, D-06120 Halle/Saale, Germany; andreas.thiel@student.uni-halle.de (A.T.); iris.thondorf@biochemtech.uni-halle.de (I.T.)

**Keywords:** breast cancer, CA IX, CA XII, CA inhibitor, betulin derivative, sulfonamides, hypoxia

## Abstract

Hypoxia-regulated protein carbonic anhydrase IX (CA IX) is up-regulated in different tumor entities and correlated with poor prognosis in breast cancer patients. Due to the radio- and chemotherapy resistance of solid hypoxic tumors, derivatives of betulinic acid (BA), a natural compound with anticancer properties, seem to be promising to benefit these cancer patients. We synthesized new betulin sulfonamides and determined their cytotoxicity in different breast cancer cell lines. Additionally, we investigated their effects on clonogenic survival, cell death, extracellular pH, HIF-1α, CA IX and CA XII protein levels and radiosensitivity. Our study revealed that cytotoxicity increased after treatment with the betulin sulfonamides compared to BA or their precursors, especially in triple-negative breast cancer (TNBC) cells. CA IX activity as well as CA IX and CA XII protein levels were reduced by the betulin sulfonamides. We observed elevated inhibitory efficiency against protumorigenic processes such as proliferation and clonogenic survival and the promotion of cell death and radiosensitivity compared to the precursor derivatives. In particular, TNBC cells showed benefit from the addition of sulfonamides onto BA and revealed that betulin sulfonamides are promising compounds to treat more aggressive breast cancers, or are at the same level against less aggressive breast cancer cells.

## 1. Introduction

Breast cancer is the most common cancer in women in developed countries [1]. Therapies include surgery, systemic chemotherapy, radiotherapy, and hormone therapy, with new drugs continuously being developed [2]. Nevertheless, cancer-specific deaths of women are, in most cases, due to breast cancer. In particular, hormone receptor- (estrogen (ER) and progesterone (PR)) and HER2-negative tumors are resistant to therapy and thus linked to poor prognosis [2]. Moreover, hypoxic areas in tumors caused by poor oxygen supply are known for their resistance to therapy. Both characteristics impose new possible focal points in the development of breast cancer therapeutics [3].

Carbonic anhydrases (CAs) are zinc metalloenzymes ubiquitously found in the animal kingdom. To date, **15** different isoforms have been described in human cells [3]. Hypoxia is a common occurrence in tumor cells, and tumors often show an increased amount of CA IX, which is regulated by hypoxia-inducible factor 1α (HIF-1α), a hypoxia-dependent transcription factor [3,4]. CA IX is a transmembrane protein with an extracellular catalytic capacity. It plays an important role in the maintenance of the physiological intracellular pH [5]. It catalyzes the hydration of CO_2_ to HCO_3_^−^ and H^+^ in the extracellular matrix [3,5], which leads to extracellular acidosis [3,5,6]. In recent years, interest in CA IX has grown, as it has become a potential point of attack in hypoxic cancer therapy. Studies have shown a significant role of CA IX in the development and growth of tumors as well as metastases [7,8]. A correlation between the expression of CA IX in cancer cells and poor survival of patients was found [7]. Another tumor-relevant carbonic anhydrase is CA XII, which is also overexpressed in different tumor entities and shows an association with prognosis [9]. These discoveries have led to the enforced development of carbonic anhydrase inhibitors (CAIs). Different groups of CAIs have been established, such as sulfonamides and sulfamates [5,10]. In particular, CAIs based on sulfonamides have shown promising results in in vitro studies [11,12]. The sulfonamide-based inhibitor acetazolamide has been in clinical use for years and is well tolerated as a diuretic and treatment for glaucoma. Additionally, the CA IX/CA XII inhibitor SCL-0111 is in phase Ib/II clinical trials for pancreatic cancer [13,14].

Betulin is a plant-derived pentacyclic triterpene found in over 200 plant species [15]. Through oxidation, betulinic acid (BA; (3β)-3-hydroxy-lup-20(29)-en-28-oic acid) is obtained [16]. The antitumor effects of betulin and BA are similar, especially their proapoptotic effects against malignant tumor cells. However, in recent years, interest in BA and betulin/BA derivatives has grown due to its greater relevance as potential cancer therapeutics so far. BA has been shown to possess antiproliferative, anti-inflammatory, and antineovascular activity [17,18,19]. It inhibits tumor growth and cell migration and leads to cell cycle arrest and apoptosis [17,19,20]. Different studies have shown the effects of BA against various types of cancer in vitro and in vivo with increased impact under hypoxic conditions [20,21,22,23]. In addition, BA has been shown to be less toxic towards normal cells than tumor cells [21]. Our group showed strong BA-induced cytotoxicity and an early increase in apoptosis in human breast cancer cells under hypoxia. Independent of oxygen level, additive effects with irradiation were observed [22]. Thus, modification of BA is an outstanding strategy for the development of BA derivatives with new properties. BA offers the possibility to generate derivatives via variable substituents at positions C3, C20 and C28 [24]. BA and its derivatives have been evaluated for tumor therapy in vitro and in vivo [25,26]. Our own recent study determined the pharmacological properties of BA derivatives NVX-207 and B10, which revealed increased effects on migration, cell death and radiosensitivity compared to BA [27]. In an in vivo study, reduction of tumor mass by NVX-207 was also reported [28]. In addition, other studies evaluated BA and betulin sulfamates as tumor agents with high cytotoxicity, which cause a dose-dependent increase in apoptosis [22,29].

**Figure 1:**
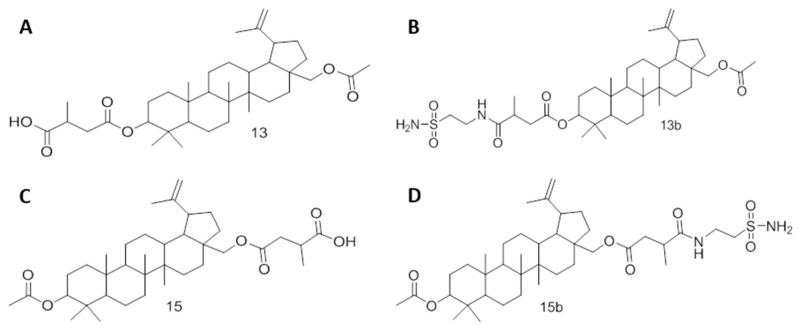
Structures of betulin derivatives. Betulin derivatives **13** (**A**) and **15** (**C**) are precursors of betulin sulfonamides **13b** (**B**) and **15b** (**D**). Although **13** and **15** contain acid groups in positions C3 and C28, respectively, **13b** and **15b** carry sulfonamide groups in these positions.

In the present study, we designed and synthesized four betulin derivatives (Figure 1). In particular, as precursor substances, betulin derivatives (**13**, **15**) were substituted with a sulfonamide (**13b**, **15b**) to combine the cytotoxic effects of betulin/betulinic acid under hypoxic conditions with the possibility of targeting the tumor-associated and hypoxia-regulated proteins CA IX and CA XII. Although precursors **13** and **15** possess acidic groups at C3 and C28, respectively, **13b** and **15b** contain sulfonamide groups at these sites. We hypothesized that the combination of betulin and sulfonamides would generate a stronger cytotoxic effect and radiosensitization in breast cancer cells due to the CA-inhibiting properties of sulfonamides in contrast to acidic groups.

## 2. Results

### 2.1. Receptor Status and Basal HIF-1α, CA IX and CA XII Expression Levels in Breast Cancer Cell Lines

**Figure 2:**
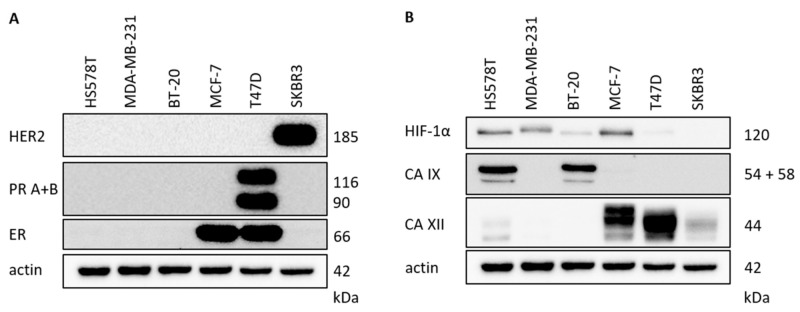
Characterization of human breast cancer cell lines (under normoxic conditions). Characterization of protein expression in six human breast cancer cell lines under normoxic conditions by use of Western blot. The detection of hormone receptors progesterone (PR A+B) and estrogen (ER) as well as HER2 (**A**). Protein levels of HIF-1α, CA IX and CA XII are shown (**B**). Actin was used as the loading control. The figure shows a representative Western blot.

Breast cancer cells can be characterized by evaluation of the receptor status of ER, PR and HER2 [30]. Proof of the receptors in the breast cancer cell lines was detected by Western blot: SKBR3 as a HER2-positive cell line, T47D as an ER- and PR-positive cell line and MCF-7 as an ER-positive cell line (Figure 2A, Table 1). In the three triple-negative breast cancer (TNBC) cell lines (HS578T, MDA-MB-231 and BT-20), HER2, PR and ER were not detectable, as expected [30] (Figure 2A, Table 1).

Additionally, the protein expression levels of HIF-1α and carbonic anhydrases CA IX and CA XII were determined (Figure 2B). All investigated cell lines showed low or intermediate normoxic HIF-1α protein levels. The breast cancer cell lines HS578T and BT-20 showed high CA IX protein levels, but in the other cell lines, CA IX was not detectable. In contrast, high CA XII protein levels were detectable in T47D and MCF-7 cells, and low CA XII protein levels were detectable in HS578T and SKBR3 cells. MDA-MB-231 and BT-20 cells do not express CA XII.

### 2.2. Hypoxia-Induced CA 9/CA IX and CA 12/CA XII Levels in Breast Cancer Cell Lines

**Figure 3:**
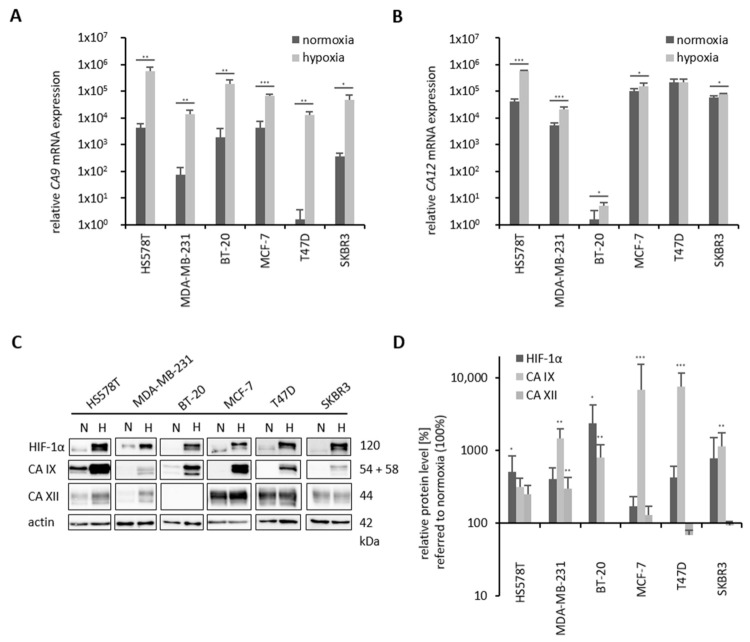
Influence of hypoxia on *CA 9* and *CA 12* mRNA and HIF-1α, CA IX, CA XII protein expression levels. Breast cancer cell lines were cultured under normoxia (21% O_2_) and hypoxia (0.1% O_2_) for 24 h. RNA and protein were isolated from the cell lysates. RT-PCR was used to determine the mRNA expression levels of *CA 9* (**A**) and *CA 12* (**B**). HIF-1α, CA IX and CA XII protein levels were verified by Western blot (**C**) and the influence of hypoxic conditions on HIF-1α, CA IX and CA XII protein levels was quantified (**D**). Values determined from the hypoxic samples of each cell line were compared to the corresponding normoxic sample, which was set as 100%. Data (mRNA or protein level) represent mean values (+SD) of at least three independent experiments. Significant *p* values are highlighted with asterisks (* *p* ≤ 0.05, ** *p* ≤ 0.01, *** *p* ≤ 0.001). In Western Blot analysis actin was used as the loading control.

In the six human breast cancer cell lines with different molecular subtypes (Figure 2, Table 1), the influence of hypoxia (0.1% O_2_) on *CA 9*/CA IX and *CA 12*/CA XII mRNA and protein expression levels was investigated (Figure 3). Under normoxic conditions (21% O_2_), T47D breast cancer cells express very little *CA 9*. MDA-MB-231 and SKBR3 cells have low *CA 9* mRNA expression compared to BT-20, MCF-7 and HS578T cells, although in HS578T cells, the highest *CA 9* mRNA expression level was detected (Figure 3A). However, under hypoxia, *CA 9* mRNA levels were significantly increased in all investigated breast cancer cell lines (Figure 3A). In contrast to the *CA 9* mRNA level (40- to 4000-fold), *CA 12* mRNA was only slightly induced by hypoxia in all cell lines (1.4- to 10-fold), except in T47D cells (Figure 3B). In the TNBC cell line BT-20, *CA 12* mRNA is almost undetectable, MDA-MB-231 cells showed a low *CA 12* mRNA level under normoxic conditions, whereas HS578T, SKBR3 and MCF-7 cells have a moderate *CA 12* mRNA level. T47D cells had the highest normoxic *CA 12* mRNA level (Figure 3B).

Investigation of protein expression revealed increased HIF-1α levels under hypoxic conditions in all investigated cell lines (Figure 3C,D). All breast cancer cell lines showed a strong increase in CA IX protein levels under hypoxic conditions by Western blot analysis (Figure 3C,D). In contrast, regulation of CA XII protein levels by oxygen depended on the cell line. In HS578T and MDA-MB-231 cells, CA XII protein levels were up-regulated under hypoxic conditions. In BT-20 cells, CA XII was not detectable under normoxic or hypoxic conditions. In the luminal breast cancer cell lines MCF-7, T47D and SKBR3, CA XII protein levels were not regulated by oxygen conditions (Figure 3C,D).

In summary, the basal TNBC cell lines (HS578T, MDA-MB-231, BT-20) showed enhanced *CA 9* mRNA and CA IX protein levels compared to luminal hormone receptor- or HER2-positive cell lines (MCF-7, T47D, SKBR3), whereas *CA 12* mRNA and CA XII protein levels were higher in these cell lines than in TNBC cell lines (Figure 3, Table 1).

### 2.3. Cytotoxicity of BA, Betulin Sulfonamides and Their Precursors in Breast Cancer Cell Lines

**Figure 4:**
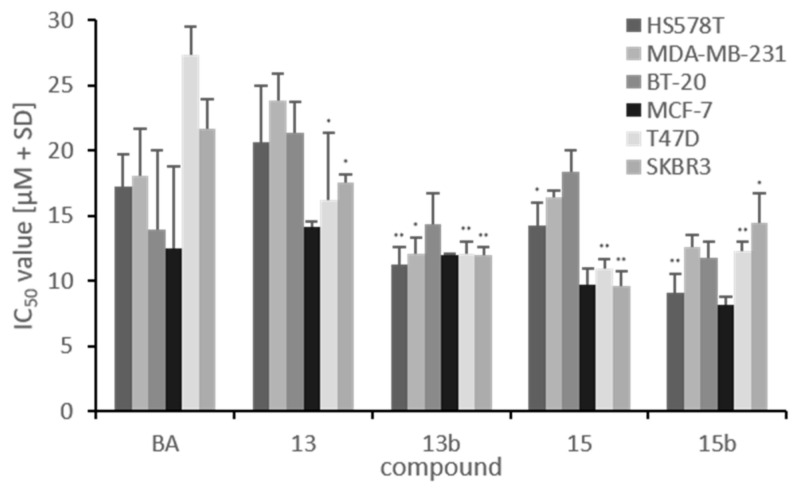
IC_50_ values [µM] of betulinic acid and betulin derivatives. IC_50_ values represent mean values (+SD) of at least three independent experiments. For each cell line, *p* values refer to the IC_50_ value of BA. Significant *p* values are highlighted with asterisks (* *p* ≤ 0.05, ** *p* ≤ 0.01).

The cytotoxicity of the tested substances was determined with an SRB assay under normoxia, and IC_50_ values are shown in Figure 4. Due to therapeutic impact of betulinic acid, we focused on the comparison of our newly synthesized betulin derivatives (**13**, **13b**, **15** and **15b**) with betulinic acid. Investigations revealed that MCF-7 was the most sensitive cell line because the IC_50_ values of the investigated betulin derivatives were in the same range (between 8 µM and 14 µM). However, precursor substances **13** and **15** were more cytotoxic than BA but only in the luminal hormone receptor- and HER2-positive cell lines (T47D and SKBR3). It is striking that the IC_50_ values of precursor substances **13** and **15** were higher in TNBC cell lines (HS578T, MDA-MB-231, BT-20) than in hormone receptor-positive (MCF-7, T47D) or HER2-positive (SKBR3) breast cancer cell lines. Additionally, modification at C28 is more cytotoxic, resulting in lower IC_50_ values for the C28-modified precursor substance 15 (10 µM–18 µM) compared to the IC_50_ values of **13** (14 µM–23 µM) in all investigated cell lines. Sulfonamide substitution leads to a strong decrease in IC_50_ values, especially in the TNBC cell lines, independent of substitution position (C3 vs. C28). The IC_50_ values of betulin sulfonamides **13b** and **15b** were in the range of 8 µM to 14 µM (Figure 4), showing a strong response from the luminal hormone receptor-positive (MCF-7, T47D), HER2-positive (SKBR3) and TNBC (HS578T, MDA-MB-231, BT-20) cell lines.

### 2.4. Effects of Betulin Sulfonamides and Their Precursors on the Clonogenic Survival of Breast Cancer Cell Lines

**Figure 5:**
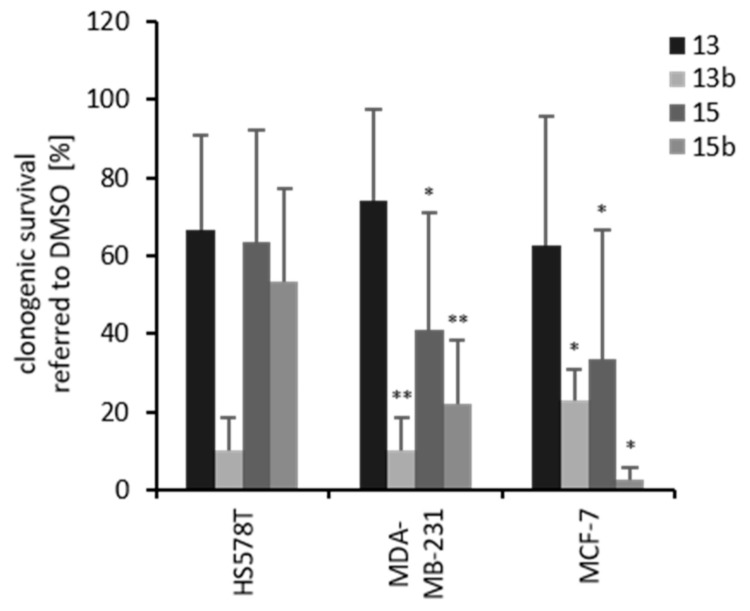
Clonogenic survival of breast cancer cells. Breast cancer cells were incubated with betulin derivatives (20 µM) under hypoxia (0.1% O_2_) for 24 h. The diagram shows clonogenic survival [%] relative to DMSO-treated cells for each breast cancer cell line. Data represent mean values (+SD) of at least three independent experiments. Significant *p* values (differences of treated cells compared to DMSO) are highlighted with asterisks (* *p* ≤ 0.05, ** *p* ≤ 0.01).

A clonogenic survival assay was performed with the breast cancer cell lines under hypoxia (Figure 5). Based on their differential basal protein expression of CA IX and CA XII, the cell lines HS578T, MDA-MB-231 and MCF-7 were selected. Furthermore, the other cell lines formed no (BT-20) or too small (T47D, SKBR3) colonies for reproducible quantification. Based on the preliminary tests, a concentration of 20 µM was selected to reliably achieve an IC_50_ value with the respective derivatives in all cell lines, and the less toxic concentration of 10 µM was selected as a comparison. Incubation of 10 µM betulin derivative (**13**, **13b**, **15** or **15b**) caused no or low effects on clonogenic survival (data not shown). However, in all investigated cell lines (HS578T, MDA-MB-231, MCF-7), betulin sulfonamides (**13b**, **15b**) caused a stronger reduction in clonogenic survival than the precursor betulin derivatives (**13**, **15**) (Figure 5). In HS578T cells, 20 µM **13b** caused the strongest reduction in clonogenic survival (10%), whereas **13**, **15** and **15b** reduced clonogenic survival to between 50% and 70%. In MDA-MB-231 and MCF-7 cells, betulin sulfonamides had stronger effects on clonogenic survival than their precursor substances. A reduction in clonogenic survival to between 3% and 23% could be achieved by treatment with **13b** or **15b**, respectively (Figure 5).

### 2.5. Induction of Cell Death through Betulin Sulfonamides and Their Precursors in Breast Cancer Cell Lines

**Figure 6:**
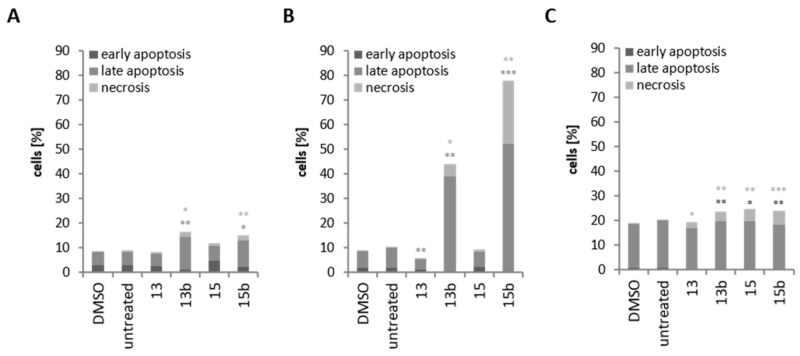
Annexin PI staining. HS578T (**A**), MDA-MB-231 (**B**) and MCF-7 (**C**) cells were treated with 20 µM **13**, **13b**, **15** and **15b** for 24 h under hypoxia (0.1% O_2_). The supernatant and cells were collected and stained with annexin V-PI. Cells stained negative for both annexin V and PI were alive. Early apoptotic cells stained positive for annexin V but negative for PI, whereas late apoptotic or dead cells stained positive for both annexin V and PI. Necrotic cells are indicated as negative for annexin V but positive for PI. Data represent mean values of at least three independent experiments. Significant *p* values are highlighted with asterisks (* *p* ≤ 0.05, ** *p* ≤ 0.01, *** *p* ≤ 0.001).

Hypoxic treatment led to the induction of cell death by approximately 9% in HS578T and MDA-MB-231 cells and 20% in MCF-7 cells (DMSO, untreated) (Figure 6). In HS578T and MDA-MB-231 cells, precursor substances **13** or **15**, respectively, did not induce cell death (Figure 6A,B), whereas in MCF-7 cells, the induction of necrosis was observed after treatment with precursors (**13**, **15**) or sulfonamides (**13b**, **15b**) (Figure 6C). However, HS578T cell treatment with **13b** or **15b** caused a slight increase in cell death, approximately 15% (Figure 6A). Treatment of MDA-MB-231 cells with **13b** or **15b** resulted in strong induction of apoptosis and necrosis. The number of apoptotic cells increased from 7% (DMSO) to 40% (**13b**) or 50% (**15b**), whereas the number of necrotic cells increased to 5% (**13b**) or 26% (**15b**), respectively (Figure 6B).

### 2.6. Molecular Docking of Betulin Sulfonamides with CA IX

**Figure 7:**
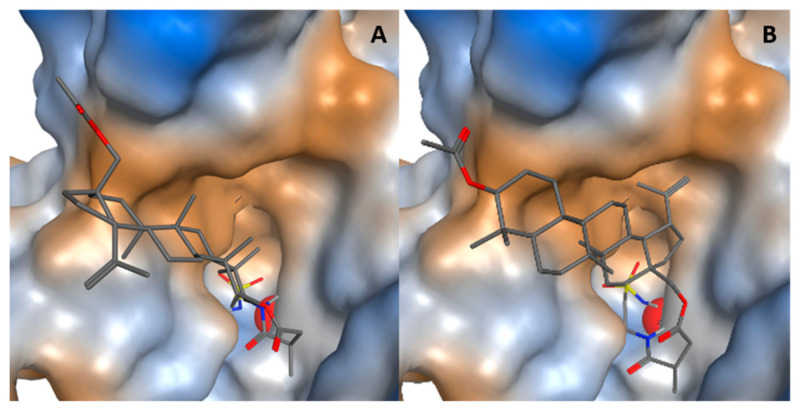
Docking poses of betulin sulfonamides **13b** and **15b** in the binding pocket of CA IX (PDB: 6g9u). The predicted binding modes of **13b** (**A**) and **15b** (**B**). The binding pocket of CA IX is colored as a molecular surface according to lipophilicity (brown: lipophilic, blue: hydrophilic, white: neutral). The zinc ion is shown as a red sphere.

Both betulin sulfonamides **13b** and **15b** bind in the binding pocket of CA IX. The sulfonamide group is bound to zinc, the spacer is in the binding pocket, and the betulin ring skeleton is in or at the edge of the binding pocket (Figure 7). Compounds **13b** and **15b** have different binding modes with respect to the betulin backbone despite their similar length and spacer structure. The different links between betulin and the spacer, C3 for **13b** and C28 for **15b**, result in different positions in the binding pocket. Inhibitor **15b** has betulin positioned deeper in the binding pocket than inhibitor **13b**. This is confirmed by ligand interaction diagrams, which illustrate the interactions of the betulin derivatives **13b** and **15b** with the active site residues of carbonic anhydrase IX, and a higher ChemPLP score of betulin sulfonamide **15b** than betulin sulfonamide **13b** (see also Figure S1 and Table S1 in the Supplementary Materials). The orientation of CA XII is consistent with that of CA IX, but the orientation of the two inhibitors in the binding pockets is very similar (see also Figure S2 in the Supplementary Materials).

### 2.7. Effects of Betulin Sulfonamides and Their Precursors on Extracellular PH Values of Breast Cancer Cells

**Figure 8:**
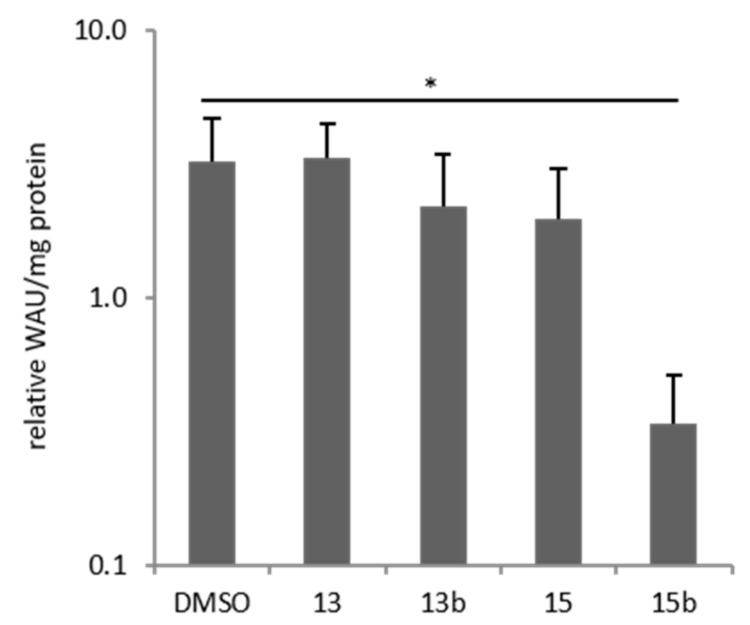
Hypoxia-induced CA activity. Extracellular pH measurements were used to determine CA activity. HS578T cells were cultured under normoxia (21% O_2_) or hypoxia (0.1% O_2_) for 24 h and subsequently treated with betulin derivatives for 3 h. The pH_e_ value of normoxic cells was set as 1.0 WAU/mg protein and the diagram shows the relative WAU/mg protein of the treated cells. Data represent mean values (+SD) of at least five independent experiments. Significant *p* values are highlighted with an asterisk (* *p* ≤ 0.05).

Membrane-bound CA IX and CA XII catalyze the hydration of carbon dioxide (CO_2_) to bicarbonate (HCO_3_^−^) and a hydrogen ion (H^+^), which leads to an acidic extracellular pH (pH_e_). Measurement of extracellular pH was therefore performed to determine the ability of betulin derivatives to inhibit hypoxia-induced CA activity in a cell-based system. Culturing breast cancer cells for 24 h under hypoxic conditions led to a significant three-fold increase in CA activity (*p* = 0.05, DMSO, Figure 8). Betulin sulfonamide **15b** was the only betulin derivative that caused a significant reduction in CA activity in HS578T cells under hypoxia (Figure 8). However, betulin derivative **13** had no effect on CA activity, whereas **13b** and **15** exhibited slight decreases.

### 2.8. Effects of Betulin Sulfonamides on HIF-1α, CA IX and CA XII Protein Expression Levels

**Figure 9:**
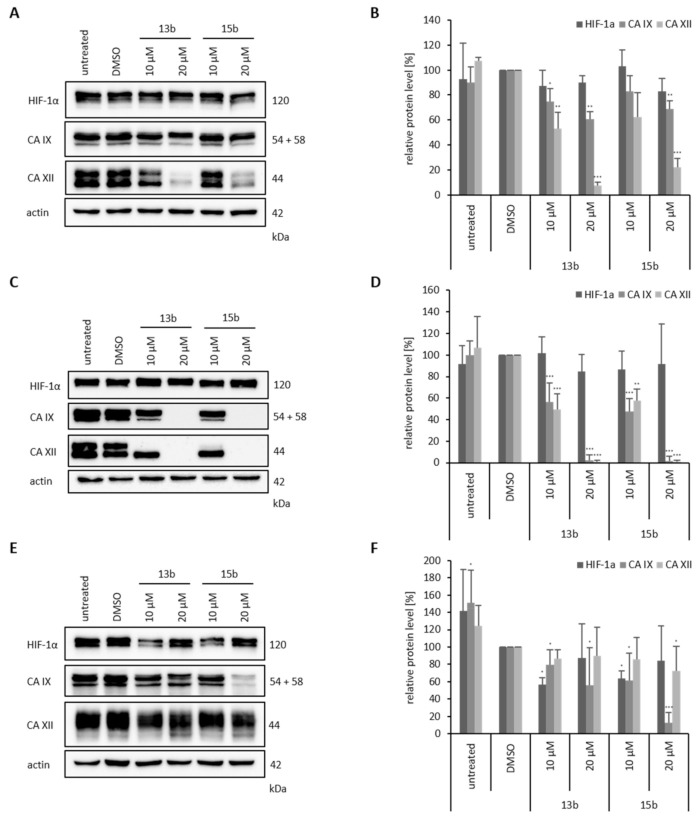
Western blots of HIF-1α, CA IX and CA XII. Three breast cancer cell lines HS578T (**A** + **B**), MDA-MB-231 (**C** + **D**) and MCF-7 (**E** + **F**) were treated with 10 µM and 20 µM **13b** and **15b** for 24 h under hypoxia (0.1% O_2_). The cell lysates were analyzed by Western blot, and the protein levels of HIF-1α, CA IX and CA XII were examined. Actin was used as a loading control. The figure shows representative Western blots (**A**,**C**,**E**), but quantification (**B**,**D**,**F**) was performed from at least three independent experiments. Significant *p* values are highlighted with asterisks (* *p* ≤ 0.05, ** *p* ≤ 0.01, *** *p* ≤ 0.001).

Additionally, we performed Western blot analysis to investigate the HIF-1α, CA IX and CA XII protein levels in the breast cancer cell lines HS578T, MDA-MB-231 and MCF-7 after treatment with the betulin sulfonamides under hypoxia (Figure 9). In all three cell lines, the protein level of HIF-1α was unchanged after treatment with **13b** or **15b**. We only observed an impact of betulin sulfonamides on CA IX and CA XII protein level after 24 h of treatment (Figure 9, see also Figure S3 in the Supplementary Materials). However, CA IX and CA XII protein levels were differentially influenced by **13b** and **15b** depending on the cell line. In HS578T cells, the level of CA IX remained almost unchanged, but CA XII protein levels decreased by 80% after treatment with 20 µM **13b** or **15b** (Figure 9A,B). In contrast, in MDA-MB-231 cells, 10 µM **13b** or **15b** caused a significant decrease in CA IX and CA XII protein levels by approximately 50%, and after treatment with 20 µM **13b** or **15b**, the levels of CA IX and CA XII were almost undetectable (Figure 9C,D). Furthermore, in the MCF-7 cell line, the CA IX level was clearly reduced after the application of 20 µM **15b**. CA XII was not affected by either **13b** or **15b** (Figure 9E,F).

### 2.9. Radiosensitivity

**Figure 10:**
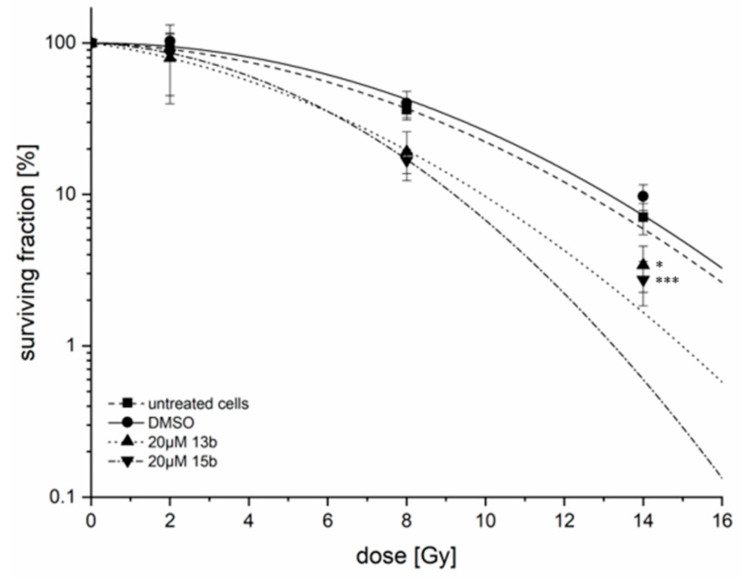
Radiosensitivity of MDA-MB-231 cells under hypoxic conditions. Cells were treated with 20 µM **13b** or **15b** for 24 h and irradiated with 0 Gy, 2 Gy, 8 Gy and 14 Gy. A clonogenic survival assay was performed 1 h after irradiation and cultured for 12 d. All colonies that consisted of more than 50 cells were counted. The diagram shows the overall survival [%] and plating efficiency of each treatment; the value at 0 Gy was set as 100%. Data represent mean values (±SD) of four independent experiments. EF_14Gy_ was 3.16 ± 1.36 for **13b** (* *p* = 0.02) and 3.70 ± 0.65 for **15b** (*** *p* < 0.001).

Since the effects of derivatives **13b** and **15b** on the protein expression of CA IX and CA XII were strongest in MDA-MB-231 cells, this cell line was selected to investigate the effects of these derivatives on radiosensitivity. Although 10 µM **13b** or **15b** reduced CA IX and CA XII protein levels by approximately 50% in MDA-MB-231 cells under hypoxic conditions (Figure 9C,D), radiosensitivity was not altered (data not shown). However, incubation of MDA-MB-231 cells with 20 µM **13b** or **15b** under hypoxic conditions caused radiosensitization of these breast cancer cells (Figure 10). For betulin derivative **13b**, a DMF_10_ of 1.25 ± 0.14 (*p* = 0.01) and an EF_14Gy_ = 3.16 ± 1.36 (*p* = 0.02), and for **15b**, a DMF_10_ of 1.36 ± 0.13 (*p* < 0.001) and an EF_14Gy_ = 3.70 ± 0.65 (*p* < 0.001) were calculated.

## 3. Discussion

BA has many anticancer properties, such as antiproliferative, proapoptotic, anti-inflammatory and antiangiogenic [25]. Due to its poor solubility, its application for therapy is limited. Therefore, the development of derivatives with similar properties, especially with increased cytotoxicity under hypoxic conditions in tumor cells, but improved solubility is the current focus of research. The combination of hypoxic cytotoxicity and inhibition of genes/proteins that are important for the adaption of metabolism due to hypoxic conditions are of particular interest, for example CAs. For instance, the synthesis of betulin sulfamates is an interesting approach for the treatment of hypoxic tumors [22,29]. We synthesized betulin derivatives that were substituted with a sulfonamide on C3 (**13b**) or C28 (**15b**) and compared the activities of the compounds with those of BA and precursors **13** and **15**. The compounds were tested for their cytotoxicity and potential to inhibit tumor-relevant processes in human breast cancer cell lines.

Consistent with the results of Chen et al. [31], we detected elevated CA IX levels in basal TNBC cell lines (HS578T, MDA-MB-231, BT-20) and high expression of CA XII in luminal hormone receptor-positive (MCF-7, T47D) or HER2-positive (SKBR3) breast cancer cell lines. Some studies have assumed crosstalk between carbonic anhydrases [32,33,34]. In detail, in the colon carcinoma cell line LS174Tr, knockdown of *CA 9* revealed up-regulation of *CA 12*, and increased CA XII expression was observed from the immunohistochemical results of tumor sections of mice injected with *CA 9*-diminished cells in vivo. [33]. Both the triple-negative phenotype and elevated CA IX expression in breast cancer are independently associated with a poor clinical outcome and resistance to different therapeutic options (radiation, chemotherapy) [7,35,36]. Although the importance of CA XII for the prognosis of cancer patients is controversial [9], high *CA 12*/CA XII expression is associated with better disease-free survival of breast cancer patients [31,37]. However, a high or elevated CA XII level is possibly a rescue mechanism due to the low or decreased CA IX levels and is responsible for better clinical outcomes. Therefore, it could be beneficial to inhibit both CA IX and CA XII, especially in TNBC, to reduce possible crosstalk between these carbonic anhydrases and consequently prevent a rescue mechanism.

Analysis of the cytotoxicity of the newly synthesized betulin derivatives **13**, **13b**, **15** and **15b** revealed that precursors **13** and **15** showed higher cytotoxicity in hormone-positive (MCF-7, T47D) or HER2-positive (SKBR3) cell lines than BA, but no increased cytotoxicity in TNBC cell lines (HS578T, MDA-MB-231, BT-20). However, betulin sulfonamides (**13b**, **15b**) were more cytotoxic than BA and their precursors in all investigated breast cancer cell lines independent of hormone or HER2 status. This could possibly be explained by the characteristic of sulfonamides to inhibit CA IX compared to the precursor substances because TNBC cell lines have an elevated basal (normoxic) CA IX expression level [38]. This means that inhibition of CA IX leads to a reduction in the resistance of TNBC to chemotherapy. Andreucci et al. showed that inhibition of CA IX and CA XII with ureido-substituted sulfamate SCL-0111, which is already in clinical trials phase II, can increase the response of different tumor types (melanoma, breast and colon cancer) to chemotherapy (dacarbazine, temozolomide doxorubicin or 5-fluorouracil) [39]. For colorectal cancer, in vitro and in vivo studies have proved abrogation of chemoresistance after combined treatment with doxorubicin and a carbonic anhydrase inhibitor. In vivo tumor growth was significantly decelerated after combined chemotherapy with a CAI in tumor-bearing mice [40,41].

However, hypoxic conditions increased CA IX levels in all investigated breast cancer cell lines (Figure 3). Nevertheless, the clonogenic survival of breast cancer cells was reduced under hypoxic conditions after treatment with the betulin derivatives but to a stronger extent after treatment with the betulin sulfonamides **13b** and **15b** (Figure 5). The decreased clonogenic survival to approximately 60% in all investigated breast cancer cell lines with precursor substances **13** and **15** was probably caused by the betulin scaffold. Previous studies have already proven the inhibitory effects of betulinic acid on clonogenic survival in human lung cancer, breast cancer and glioma cells, especially under hypoxic conditions [22,23,42]. Moreover, an inhibitory effect on clonogenic survival was also shown for different tumor cells with reduced *CA 9* expression or CA IX activity [6,22,43,44]. Therefore, the combination of the betulin scaffold and the CA IX-inhibiting sulfonamide group (in **13b** and **15b**) reinforces the inhibitory effect on clonogenic survival compared to the precursors **13** and **15** in breast cancer cell lines.

Investigation of cell death under hypoxic conditions with annexin V-PI staining revealed almost no influence of betulin derivatives **13** and **15** in any of the three cell lines investigated (MDA-MB-231, HS578T and MCF-7). However, in HS578T and MDA-MB-231 cells, application of **13b** or **15b**, respectively, induced cell death, especially apoptosis and necrosis, which was seen to a greater extent in MDA-MB-231 cells (Figure 6). Although both cell lines displayed similar IC_50_ values, a possible reason for the lesser induction of apoptosis in HS578T cells could be a concentration- and time-dependent effect. In contrast, in MCF-7 cells, only the induction of necrosis, but not apoptosis, was detectable after treatment with betulin derivatives **13b** and **15b**. An earlier study showed that betulinic acid and different betulin sulfamates induce apoptosis in a time-dependent manner in MDA-MB-231 cells and that in MCF-7 cells, only low induction of apoptosis could be observed [22]. A possible explanation for the absence of induction of apoptosis is that MCF-7 cells are defective in caspase-3 [45]. However, in other tumor entities, such as prostate, colorectal, renal or cervical cancer, inhibition of CA IX activity causes apoptosis as well [46,47,48]. One major player of induction of apoptosis are mitochondria. The loss of mitochondrial membrane potential followed by release of cytochrome c to cytoplasm due to permeabilization of outer mitochondrial membrane can stimulate apoptosis [49]. Betulin, betulinic acid and their derivatives can influence the membrane of cells and organelles, especially mitochondria. Several studies show a decrease of mitochondrial membrane potential (∆_Ψ_), an inhibition of the activity of respiratory chain complexes and initiation of ROS (reactive oxygen species) formation due to treatment with betulin, betulinic acid or their derivatives [50,51,52]. Detailed investigations of the distinct mechanisms of cell death initiated by **13b** and **15b** should be elucidated in future studies.

The most important impact of carbonic anhydrase activity (CA IX and CA XII) is the contribution of the maintenance of intracellular pH to ensure the survival of tumor cells through the accumulation of acidic metabolic products (lactic acid, CO_2_). Cells lacking CA IX are characterized by a low intracellular pH when exposed to acidic medium and diminished cell proliferation. However, stable transfection of *CA 9* stabilizes the intracellular pH and favors cell survival under acidic cell culture conditions [33]. McIntyre also showed a decreased intracellular pH after inhibition of different bicarbonate transporters, which resulted in the induction of apoptosis and necrosis, proving the impact of intracellular pH on cancer cell survival [34].

The reduced impact of carbonic anhydrases could be due to decreased protein levels or diminished activity. We have proven for the potential CAI **13b**, there is a slight, and for **15b**, there is a strong significant reduction in hypoxia-induced CA activity (Figure 8) by measuring the changes of extracellular pH value. The minor impact of **13b** on CA activity compared to **15b** could be explained by the more advantageous positioning of C28-substituted betulin sulfonamide **15b** in the binding pocket of CA IX compared to the C3-substituted betulin sulfonamide **13b** (Figure 7). In contrast to 24 h treatment with **13b** and **15b** (Figure 9), no impact of betulin sulfonamides was observed after 3 h of incubation on CA IX and CA XII protein level (see also Figure S3 in the Supplementary Materials). The reduction of hypoxia-induced CA activity by **13b** and **15b** is not due to reduced protein level. However, Western blot analysis after 24 h of incubation revealed that both betulin sulfonamides **13b** and **15b** caused a decrease in CA IX and/or CA XII protein levels in all three cell lines tested (MDA-MB-231, HS578T, MCF-7) (Figure 9). The decrease in membrane-bound carbonic anhydrases may be explained by different mechanisms that may be involved in the abundance of CA IX and CA XII, especially in MDA-MB-231 cells [53]. Endocytosis is an important process that can be induced by stress conditions and regulates the abundance of membrane-bound proteins/receptors [54]. Additionally, for CA IX, so-called ectodomain (ECD) shedding has been described, which converts membrane-bound molecules into soluble molecules. ECD shedding can be induced by chemical compounds or drugs that are responsible for the induction of cell death [54]. In CGL3 hybrid cells, doxorubicin treatment led to the induction of apoptosis and a reduction in membrane-bound CA IX as well as elevated CA IX shedding, which was mediated by metalloproteinases. The application of the broad metalloproteinase inhibitor batimastat inhibited apoptosis-associated shedding [55]. In accordance with our results, Hektoen et al. detected a decrease in CA IX protein levels after incubation with the CA IX inhibitor S4 but an increase in soluble CA IX protein in the cell culture medium [56].

CA IX and CA XII expression is regulated by HIF-1α because of a hypoxia-responsive element (HRE) in the promotor region of both *CA 9* and *CA 12* [57,58]. Despite the decrease in CA IX and CA XII protein levels after treatment with **13b** and **15b** in breast cancer cells, HIF-1α protein levels were not altered. However, an AP-1-responsive element and potential NF-κB binding sites in the *CA 9* promotor region have been described and may contribute to the regulation of *CA 9* transcription [59,60]. Andreucci et al. described an alternative, HIF-1α-independent regulation mechanism of CA IX under acidic conditions through NF-κB in cancer cells. Acidic pH, which is characteristic of hypoxia, induces NF-κB [61,62] and increases *CA 9* expression, which is repressed by the NF-κB inhibitor parthenolide in human melanoma cells [63]. The CAI-mediated reduction in CA IX is possibly triggered by NF-κB. It is conceivable that the increase in extracellular pH after treatment with CAIs [47,64] diminishes the activity of NF-κB, which conversely decreases *CA 9*/IX expression. Further investigations concerning the distinct mechanisms responsible for the abundance of membrane-bound CA IX are necessary, specifically concerning the internalization of CA IX and CA XII after treatment and the influence of betulin sulfonamides on the activity of NF-κB and resulting regulation of *CA 9*/CA IX expression.

For various tumor entities (breast cancer, renal cell cancer), a radiosensitization effect from pharmacological inhibition and silencing of *CA 9* has been proven in different studies [6,22,65]. This is in accordance with our observation of the strong radiosensitization of MDA-MB-231 cells after treatment with **13b** or **15b**, which both inhibit CA IX activity and/or CA IX and CA XII protein levels (Figure 10). Doyen et al. showed that knockdown of *CA 12* radiosensitizes colon carcinoma cells. In accordance with our data, simultaneous knockdown of *CA 9* and *CA 12* in colon carcinoma cells resulted in stronger radiosensitization than single knockdown of either *CA 9* or *CA 12* [66], which is possibly due to a rescue mechanism between CA IX and CA XII [33]. There is evidence that radiosensitization is also triggered by the pH of the extracellular microenvironment [47,64]. Acidic culture conditions lead to radiosensitization of *CA 9*-depleting fibroblasts because maintenance of the intracellular pH is restricted [66].

## 4. Materials and Methods

### 4.1. Preparation of Precursors—Compounds ***13*** and ***15***

**Figure 11:**
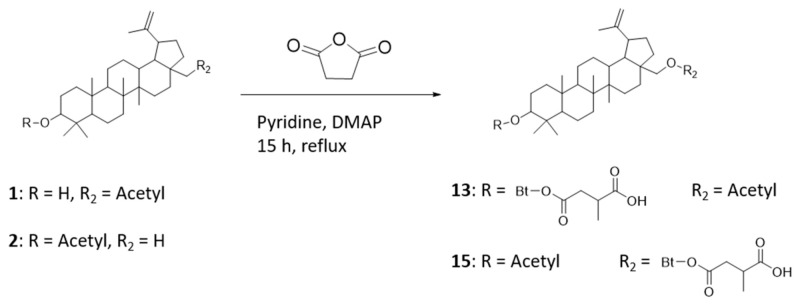
Preparation of 4-((1R)-3a-(acetoxymethyl)-5a,5b,8,8,11a-pentamethyl-1-(prop-1-en-2-yl)icosahydro-1H-cyclopenta[a]chrysen-9-yloxy)-2-methyl-4-oxobutanoic acid (compound **13**) and 4-(((1R)-9-acetoxy-5a,5b,8,8,11a-pentamethyl-1-(prop-1-en-2-yl)icosahydro-1H-cyclopenta[a]chrysen-3a-yl)methoxy)-2-methyl-4-oxobutanoic acid (compound **15**).

Compound **1** or **2** (1 g, 1.7 mmol) was dissolved in dry pyridine (10 mL), and a catalytic amount of 4-dimethylaminopyridine (DMAP) was added (Figure 11). Succinic anhydride, also dissolved in dry pyridine (5 mL), was then added, and the reaction mixture was refluxed for 15 h. After completion of the reaction, the mixture was added to ice-cold water (500 mL), and the product was extracted several times with dichloromethane. The combined organic phases were washed first with 5% HCl, then with saturated NaHCO_3_ and finally with H_2_O. After drying over Na_2_SO_4_, the solvent was removed, and the crude product was purified via column chromatography with dichloromethane (DCM)/methanol (15:1).

Characterization of **13**: ^1^H-NMR: (400 MHz, DMSO-d6): 4.67 (d, 1H, C29H), 4.54 (d, C29H), 4.35 (dd, 1H, ^3^*J*H-H = 11.1 Hz + 4.8 Hz, C3H), 4.23 (d, 1H, ^2^*J*H-H = 11.1 Hz, C28H), 3.73 (d, 1H, ^2^*J*H-H = 11.1 Hz, C28H), 2.68 (m, 1H, C3′H), 2.54 (m, 1H, C2′H), 2.43 (m, 1H, C19H), 2.34 (m, 1H, C2′H), 1.99 (s, 3H, C2″H3), 1.08 (d, 3H, ^3^*J*H-H = 7.2 Hz, C4′H3), 1.94–0.76 (42H).

Characterization of **15**: ^1^H-NMR: (400 MHz, DMSO-d6): 4.67 (d, 1H, ^2^*J*H-H = 1.4 Hz, C29H), 4.53 (d, 1H, C29H), 4.34 (dd, 1H, ^3^*J*H-H = 11.1 Hz + 4.8 Hz, C3H), 4.23 (d, 1H, ^2^*J*H-H = 11.0 Hz, C28H), 3.74 (d, 1H, ^2^*J*H-H = 11.0 Hz, C28H), 2.69 (m, 1H, C3′H), 2.56 (m, 1H, C2′H), 2.41 (m, 1H, C19H), 2.38 (m, 1H, C2′H), 1.96 (s, 3H, C2″H3), 1.08 (d, 3H, ^3^*J*H-H = 7.0 Hz, C4′H3), 1.94–0.77 (42H).

### 4.2. Preparation of Betulin Sulfonamides—Compounds ***13b*** and ***15b***

**Figure 12:**
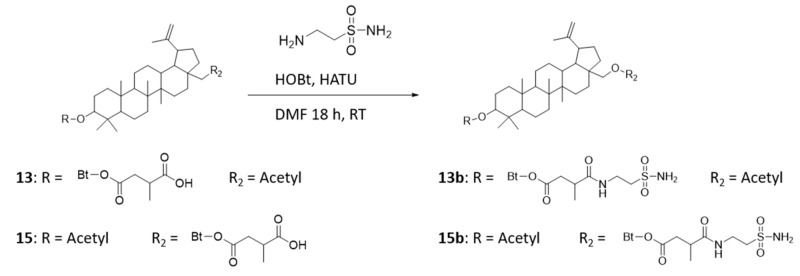
Preparation of (1R)-3a-(acetoxymethyl)-5a,5b,8,8,11a-pentamethyl-1-(prop-1-en-2-yl)icosahydro-1H-cyclopenta[a]chrysen-9-yl 3-methyl-4-oxo-4-(2-sulfamoylethylamino)butanoate (compound **13b**) and (((1R)-9-acetoxy-5a,5b,8,8,11a-pentamethyl-1-(prop-1-en-2-yl)icosahydro-1H-cyclopenta[a]chrysen-3a-yl)methyl 3-methyl-4-oxo-4-(2-sulfamoylethylamino)butanoate) (compound **15b**).

Compound **13** or **15** (200 mg, 0.35 mmol), taurine amide (87.5 mg, 0.7 mmol) and ethylene diamine (150 µL, 109 mg, 1 mmol) were dissolved in dry dichloromethane (10 mL) (Figure 12). The reaction mixture was stirred overnight at RT, the solvent was removed, and the product was diluted with ethyl acetate and washed with water. After drying over Na_2_SO_4_, the solvent was removed, and the crude product was purified via column chromatography with DCM/methanol (15:1).

Characterization of **13b**: ^1^H-NMR: (400 MHz, DMSO-d6): 8.00 (t, 1H, ^3^*J*H-H = 5.1 Hz, CONH), 6.84 (s, 2H, NH2), 4.67 (d, 1H, C29H), 4.54 (d, 1H, C29H), 4.33 (dd, 1H, ^3^*J*H-H = 11.3 Hz + 4.6 Hz, C3H), 4.23 (d, 1H ^2^*J*H-H = 11.1 Hz, C28H), 3.73 (d, 1H, ^3^*J*H-H = 11.0 Hz, C28H), 3.37 (m, 2H, C6′H2), 3.05 (t, 2H, ^3^*J*H-H = 7.2 Hz, C7′H2), 2.62 (m, 1H, C3′H), 2.55 (m, 1H, C2′H), 2.43 (m, 1H, C19H), 2.25 (m, 1H, C2′H), 1.99 (s, 3H, C2″H3), 1.01 (d, 3H, ^3^*J*H-H = 6.8 Hz, C4′H3), 1.94–0.74 (42H).

Characterization of **15b**: ^1^H-NMR: (400 MHz, DMSO-d6): 8.02 (t, 1H, ^3^*J*H-H = 5.6 Hz, CONH), 6.84 (s, 2H, NH2), 4.68 (d, 1H, ^2^*J*H-H = 1.5 Hz, C29H), 4.54 (d, 1H, C29H), 4.35 (dd, 1H, ^3^*J*H-H = 11.2 Hz + 4.8 Hz, C3H), 4.22 (m, 1H, C28H), 3.73 (m, 1H, C28H), 3.38 (m, 2H, C6′H2), 3.05 (t, 2H, ^3^*J*H-H = 7.2 Hz, C7′H2), 2.61 (m, 1H, C3′H), 2.58 (m, 1H, C2′H), 2.42 (m, 1H, C19H), 2.29 (m, 1H, C2′H), 1.97 (s, 3H, C2″H3), 1.01 (d, 3H, ^3^*J*H-H = 6.7 Hz, C4′H3), 1.90–0.78 (42H).

### 4.3. Cell Culture Conditions and Treatment of Cells

The investigated human breast cancer cell lines MDA-MB-231, HS578T, SKBR3, T47D, and BT-20 were obtained from Prof Dittmer (Clinic for Gynecology, Martin Luther University Halle-Wittenberg, Germany). The MCF-7 breast cancer cell line was purchased from CLS (Cell Lines Service GmbH, Eppelheim, Germany). Cell line authentication was achieved by short tandem repeat (STR) DNA profiling to detect possible cross-contamination between cell lines. For cultivation at 37 °C and 5% CO_2_, RPMI 1640 medium (Thermo Fisher Scientific, Waltham, MA, USA) containing L-glutamine and 25 mM HEPES was used. Additives were 10% fetal bovine serum (Capricorn Scientific, Ebsdorfergrund, Germany), 1% pyruvate (Gibco, Thermo Fisher Scientific, Waltham, MA, USA), and 2% penicillin/streptomycin (Sigma–Aldrich, St. Louis, MO, USA). Cell cultures were routinely tested for mycoplasma contamination by PCR. All analyses were conducted in the logarithmic growth phase of the cells. Cells were seeded 24 h prior to treatment in 6 well plates (Greiner Bio-One, Kremsmünster, Austria) or 96 well plates (Techno Plastic Products, Trasadingen, Switzerland), depending on the analysis to be conducted. For analyses under hypoxic conditions, GasPacks (BD, Franklin Lakes, NJ, USA) were applied that generate an oxygen level of approximately 0.1%. The examined substances BA, **13**, **13b**, **15** and **15b** were dissolved in dimethyl sulfoxide (DMSO, Sigma-Aldrich) to a final concentration of 20 mM (stock solution). Irradiation was performed with an Oncor Impression IMRT (Siemens, Munich, Germany) at a dose rate of 2 Gy/min after 24 h of incubation.

### 4.4. Quantitative Real-Time RT-PCR (QRT-PCR)

Breast cancer cell lines were cultured under normoxia (21% O_2_) and hypoxia (0.1% O_2_). After 24 h, the cells were lysed, and mRNA was isolated with Quick-RNA Miniprep as recommended by the manufacturer (ZymoResearch, Irvine, CA, USA). cDNA synthesis and qRT-PCR were performed as described previously [67]. Primers used for qRT-PCR were purchased from Sigma–Aldrich, and the sequences are shown in Table 2. POLR2A was used as a housekeeping gene and was identified as the best reference gene by Normfinder [68]. No template control was used as a negative control. For quantification of RT-PCR data, the delta delta Ct method (ΔΔCt) was used [69]. ΔCt was calculated as the difference in the Ct value of the gene of interest and the Ct value of the reference gene (POLR2A). The average Ct value of the normoxia-cultured cell line with the highest ΔCt was chosen as the calibrator sample. This was T47D for CA9 mRNA expression and BT-20 for CA12 mRNA expression. ΔΔCt was defined as the difference in ΔCt of the treated probe and ΔCt of the calibrator probe. 2^−ΔΔCt^ displays the fold change of the mRNA expression level of the treated sample to the averaged calibrator sample.

### 4.5. Annexin V-Propidium Iodide Staining

Cells were seeded 24 h before treatment. Substances **13**, **13b**, **15**, and **15b** were applied at both 10 µM and 20 µM each. After 24 h of incubation under hypoxic conditions, a staining process with FITC-annexin V and propidium iodide (PI) (BioLegend, San Diego, CA, USA) was conducted. For FACS analysis, 100,000 cells/mL were resuspended in 100 µL of annexin binding buffer (10 mM HEPES, 140 mM NaCl, 2.5 mM CaCl_2_) and then labeled with 5 µL of annexin V-FITC (BioLegend) and 1 µL of 100 µg/mL propidium iodide. Control groups (DMSO-treated cells) consisted of unstained cells, cells stained with only annexin V-FITC or PI, and cells stained with both substances. For fluorescence detection, a BD LSRFortessa (BD Biosciences, San Jose, CA, USA) with BD FACSDiva software (version 6.1.3, BD Biosciences) was used. Assessment declared annexin V- and PI-positive cells as late apoptotic, annexin V-positive and PI-negative cells as early apoptotic, annexin V-negative and PI-positive cells as necrotic, and cells negative for both staining solutions as vital.

### 4.6. Sulforhodamine B Assay

Starting 24 h after seeding in 96-well plates, cells were treated with substances BS, **13**, **13b**, **15**, and **15b** at concentrations between 0 µM and 100 µM under normoxic conditions for 96 h. Following incubation, cells were fixed with 10% trichloroacetic acid (Carl Roth GmbH, Karlsruhe, Germany). After a wash step with ice water, a solution of 0.4% sulforhodamine B (Sigma–Aldrich) was used for staining. Washing steps with 1% acetic acid (Carl Roth GmbH) were performed before drying. After dissolving in 300 µL of 20 mM Trizma base (Sigma–Aldrich), the extinction at 540 nm was measured on a TECAN GENios FL TWT (Tecan Treading AG, Männedorf, Switzerland). Calculation of IC_50_ (half-maximal inhibitory concentration) values was determined by dose response curve fitting using Origin 2019 (OriginLab Corp., Northampton, MA, USA).

### 4.7. Clonogenic Survival Assay and Radiosensitivity

Seeded 24 h before treatment in 6 well plates, cells were subsequently incubated with substances **13**, **13b**, **15**, and **15b** at concentrations of 10 µM and 20 µM under normoxic and hypoxic conditions. After 24 h of incubation, the cells were trypsinized, counted, and seeded in small quantities in 50 mL cell culture flasks. Approximately 12 days after seeding, the grown colonies were fixated and stained with Giemsa solution (Sigma–Aldrich). A minimal size of approximately 50 cells was set to be counted as a colony. For determination of radiosensitivity, cells were irradiated 24 h after incubation with **13b** or **15b**. Depending on the cell line and oxygen level, radiation doses between 2 Gy and 14 Gy were used. The survival fraction was defined as the plating efficiency of treated cells (betulin sulfonamide-treated or irradiated) compared to the plating efficiency of untreated cells (DMSO or nonirradiated, respectively). Additionally, the dose modifying factor (DMF_10_) was calculated as the quotient of the radiation doses resulting in 10% survival of treated or untreated cells. The enhancement factor (EF_14Gy_) is the ratio of the survival fraction of treated cells and control cells. Cell survival curves were fitted to a linear quadratic model (−lnS = αD + βD2) using Origin 2019.

### 4.8. Western Blot

Western blotting was performed as described previously [6]. For analysis of protein levels, following 24 h of incubation, cells were scraped off with cell lysis buffer (Cell Signaling Technology, Inc., Danvers, MA, USA) supplemented with protease inhibitor (Cell Signaling). After homogenization by ultrasound, the protein concentration was determined with the Bradford method (Bio-Rad Laboratories, Inc., Hercules, CA, USA). SDS gels (10% and 12%) were used to separate proteins by gel electrophoresis. Afterwards, the proteins were transferred to polyvinylidene fluoride (PVDF) membranes (Merck Millipore, Burlington, MA, USA) using a tank blot system (Bio-Rad Laboratories). Membranes were blocked with 10% nonfat milk/TBST (150 mM NaCl, 50 mM Tris-HCl pH 7.5, 0.1% Tween-20) and incubated with primary antibodies against CA IX (1:2000; clone no. M75; Bioscience Slovakia, Bratislava, Slovak Republic), CA XII (1:2000; Cell Signaling), HIF-1α (1:2000; BD Biosciences), HER2 (1:2000, Cell Signaling), estrogen (1:1000; Cell Signaling), progesterone (1:1500; Cell Signaling), and actin (1:5000; Sigma). We used HRP-conjugated secondary antibodies (goat anti-rabbit; 1:5000; Dako Deutschland GmbH, Hamburg, Germany; rabbit anti-mouse; 1:5000; Cell Signaling). An ECL detection system (Advansta Inc., San Jose, CA, USA) was used to visualize immune complexes with a ChemiDoc Imaging System (Bio-Rad). For stripping, the membrane was placed in stripping solution (62.5 mM Tris-HCl pH 6.8, 2% SDS, 0.75% β-mercaptoethanol) und incubated at 50 °C using a hybridization oven for 20 min. Afterwards membrane was blocked again and reprobed with primary antibodies. For quantification of protein bands, ImageJ 1.53 software was used. The relative protein levels were determined as the quotient of the gene of interest and loading control (actin protein level).

### 4.9. Extracellular PH Analysis—Measurement of CA IX Activity

Extracellular pH analysis was used to determine the CA IX activity of the breast cancer cells. Measurement of CA IX activity was performed as described previously [6]. HS578T cells were incubated with 20 µM **13**, **13b**, **15** or **15b** for 3 h. CA IX activity was calculated according to the Wilbur-Anderson method (WAU/mg = 2× (T0-T)/T*mg protein). The duration (T) to lower the pH of the isotonic buffer from 8.0 to 6.6 at 4 °C was determined [T0, unanalyzed reaction (isotonic buffer); T, catalyzed reaction (e.g., normoxia, hypoxia)].

### 4.10. Molecular Docking Simulations

Betulin derivatives **13b** and **15b** were built in MOE 2018 (Molecular Operating Environment, 2018; Chemical Computing Group ULC, 1010 Sherbrooke St. West, Suite #910, Montreal, QC, Canada, H3A 2R7) and minimized with the AMBER 10 EHT force field. The crystal structure of betulinic acid (CSD-ID: HEHPAN) from the Cambridge Structural Database was used and modified at the corresponding positions [70,71]. Molecular docking was performed with GOLD version 5.7.2 of the CSD suite version 3.1 [72]. For this purpose, the crystal structure of CA IX (PDB ID: 6g9u) from the Protein Data Bank was used [73,74]. Missing hydrogen atoms were added to the crystal structure, and the water molecules were removed. The positions of certain side chains were manually cured to remove clashes and to form hydrogen bonds. The sulfonamide group of the bound inhibitor was used as a substructure. The final structures were selected using the ChemPLP score and visual inspection.

### 4.11. Statistical Analysis

All data represent the mean value and standard deviation (+ SD) of at least three independent experiments. The significance of differences was assessed using unpaired two-sided Student’s *t*-test. A *p*-value was considered to indicate a significant difference in reference to the population of negative control cells (DMSO), if not otherwise indicated. Significant *p* values are highlighted with asterisks (* *p* ≤ 0.05, ** *p* ≤ 0.01, *** *p* ≤ 0.001).

## 5. Conclusions

The newly synthesized betulin sulfonamides **13b** and **15b** show high cytotoxicity, especially in TNBC cell lines, antiproliferative and proapoptotic effects, inhibition of clonogenic survival and radiosensitization of human breast cancer cells. Although **13b** is not as effective as an inhibitor of CA activity, both betulin sulfonamides **13b** and **15b** influence processes that regulate the abundance of membrane-bound CA IX and CA XII. In summary, substitution of sulfonamides onto the betulin scaffold is a promising strategy to regulate carbonic anhydrases and therefore, an applicable anticancer therapy for human breast cancer, especially TNBC.

## Figures and Tables

**Table 1:** Characterization of breast cancer cell lines.

Cell Line	Subtype	ER Status	PR Status	HER2 Status	HIF-1α Protein Expression	CA IX Protein Expression	CA XII Protein Expression
HS578T	basal B	−	−	−	intermediate	strong	weak
MDA-MB-231	basal B	−	−	−	intermediate	none	none
BT-20	basal A	−	−	−	weak	strong	none
MCF-7	luminal	+	−	−	intermediate	none	strong
T47D	luminal	+	+	−	weak	none	strong
SKBR3	luminal	−	−	+	none	none	weak

ER: estrogen receptor; PR: progesterone receptor; CA IX: carbonic anhydrase IX; CA XII: carbonic anhydrase XII; +/− positive/negative status.

**Table 2:** PCR primer sequences.

Gene		Position	Sequence	Product Length
*CA 9 (NM_001216.3)*	sense	742–762	5′-CGGAGCACACTGTGGAAGGCC-3′	129 bp
	antisense	870–851	5′-CCAGAAAGGCGGCCAACACG-3′	
*CA 12 (NM_001218.5)*	sense	496–513	5′-AACCCGAATGACCCGCAC-3′	101 bp
	antisense	596–575	5′-GCGTCAGGATAAAGGTCTGAGT-3′	
*POLR2A (NM_000937.5)*	sense	1371–1390	5′-CTTGCCCCGTGCCATGCAGA-3′	83 bp
	antisense	1453–1434	5′-CTCGCACCCGGCCTTCCTTG-3′	

*CA 9*, carbonic anhydrase 9; *CA 12,* carbonic anhydrase 12; *POLR2A*, DNA-directed RNA polymerase II subunit A, bp, base pair.

## Data Availability

The data presented in this study are available on request from the corresponding author.

## Supplementary Materials

The following are available online at https://www.mdpi.com/article/10.3390/ijms22168808/s1, Figure S1: Ligand interaction diagram, Figure S2: Illustration of possible docking poses for **13b** (A) and **15b** (B) in the binding pocket CA XII (PDB code: 1jd0), Figure S3: Western blots of CA IX and CA XII, Table S1: Total ChemPLP fitness values of the docked ligands **13b** and **15b**. References [75,76] are cited in the Supplementary Materials.
