# Supplementary material for: Betulin Sulfonamides as Carbonic Anhydrase Inhibitors and Anticancer Agents in Breast Cancer Cells
Source: Int J Mol Sci. 2021 Aug 16;22(16):8808. doi: 10.3390/ijms22168808 (PMC8395940; doi:10.3390/ijms22168808)
Supplement: Supplementary file 1 [file ijms-22-08808-s001.zip › ijms-1282688-supplementary.pdf]

Supplementary Files

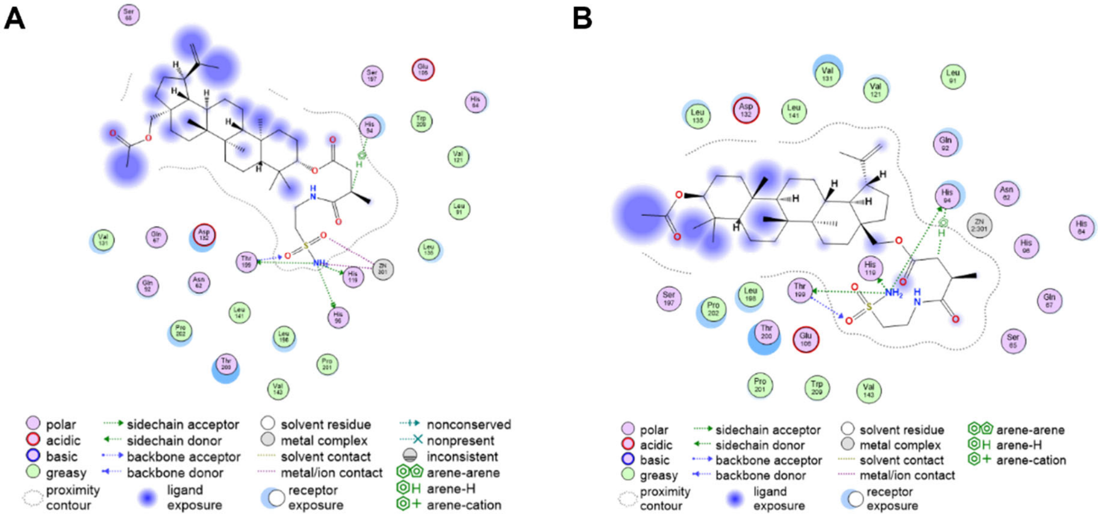

Supplementary Figure S1. Ligand interaction diagram [1]. It was generated with MOE 2020 (Molecular Operating Environment, 2018; Chemical Computing Group ULC, 1010 Sherbrooke St. West, Suite #910, Montreal, QC, Canada, H3A 2R7) illustrating the interactions of the betulin derivatives 13b **(A)** and 15b **(B)** with the active site residues of CA IX.

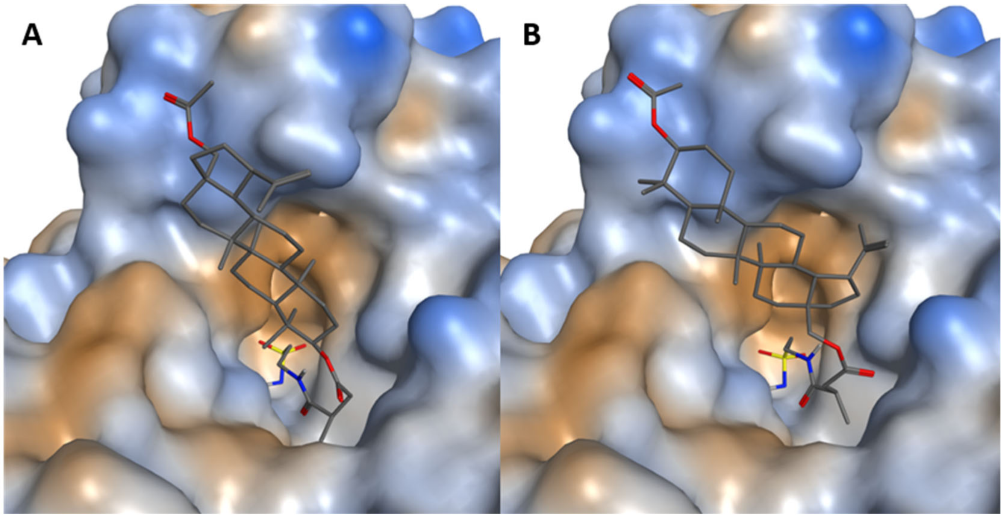

Supplementary Figure S2. Illustration of possible docking poses for 13b (**A**) and 15b (**B**) in the binding pocket CA XII (PDB code: 1jd0). The orientation of the enzyme is consistent with that of CA IX in Figure 7 of the main section. It can be seen that the orientation of the two inhibitors in the binding pockets is very similar. The binding pocket of CA XII is colored as a molecular surface according to lipophilicity (brown: lipophilic, blue: hydrophilic, white: neutral).

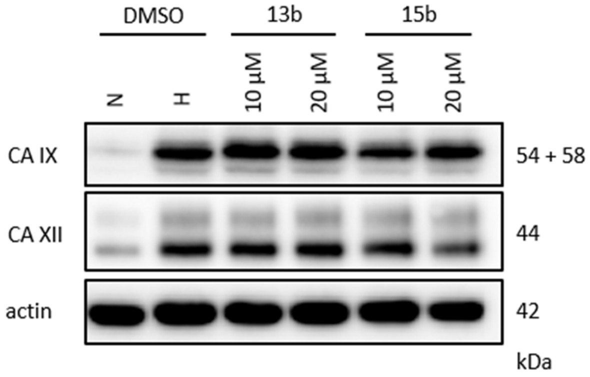

Supplementary Figure S3. Western blots of CA IX and CA XII. HS578T breast cancer cells were cultured under hypoxic conditions (0.1 % O_2) and were subsequently treated with 10 µM and 20 µM 13b and 15b for 3 h. The cell lysates were analyzed by Western blot and the protein levels of CA IX and CA XII were examined. Actin was used as a loading control. The figure shows one representative Western blots of four independent experiments.

Supplementary Table S1. Total ChemPLP fitness values of the docked ligands 13b and 15b [2]. The score values represent an empirical fitness function optimized for pose predictions of the GOLD molecular docking program. The values given are dimensionless and higher values indicate higher predictive power.

	**13b**	**15b**
Gold.PLP.Fitness	65.5	82.7

References

1. Clark, A.M.; Labute, P. 2D depiction of protein-ligand complexes. *Journal of Chemical Information and Modeling* **2007**, *47*, 1933–1944, doi:10.1021/ci7001473.
2. Korb, O.; Stützle, T.; Exner, T.E. Empirical scoring functions for advanced protein-ligand docking with PLANTS. *Journal of Chemical Information and Modeling* **2009**, *49*, 84–96, doi:10.1021/ci800298z.
